# THE PSYCHOSOMATIC SYMPTOM CLUSTERS OF OLDER ADULTS WITH DISABILITIES IN RURAL CHINA: A NATIONAL NETWORK ANALYSIS

**DOI:** 10.1093/geroni/igae098.1788

**Published:** 2024-12-31

**Authors:** Mo Yi, Zhi-wen Wang

**Affiliations:** Peking University, Beijing, Beijing, China (People’s Republic); Peking University Health Science Center, Beijing, Beijing, China (People’s Republic)

## Abstract

**Background:**

Mental health is a crucial aspect of successful aging and healthy ageing. The number and severity of older adults with disabilities (OAD) in China have been on the rise year by year. Due to impaired daily activities and reduced social participation, OAD in rural areas are more prone to various symptoms. Therefore, this study aims to construct a network of psychosomatic symptoms among OAD, identify core symptom.

**Methods:**

The study participants were 3,939 OAD aged 60 and above, sourced from the 2020 China Health and Retirement Longitudinal Study. We employed CES-D-10 scale, MMSE, and sleep-related items to assess interested outcomes, respectively. Depression was measured using 10 symptom subtypes, MMSE included six subtypes such as executive function, language function and calculation ability, etc. The sleep was categorized into three subtypes: excessive sleep, insufficient sleep, and daytime napping. “Expected Influence” and “Bridge Expected Influence” were analyzed as centrality indicators in the symptom network.

**Results:**

Network analysis results revealed that the most severe symptoms among OAD were low mood (r=1.53), impaired attention ability (r=0.98), and insufficient nighttime sleep (r=0.96). The centrality analysis identified the top symptoms as lack of vitality (r=1.06). Stratified regression analysis indicated that sleep disturbances had the greatest impact on the life satisfaction, accounting for 27.5% of the model’s explanatory power.

**Conclusion:**

Central symptoms (low mood, impaired attention and calculation ability, and insufficient nighttime sleep) and key bridge symptoms (lack of vitality) can serve as potential targets for interventions aimed at rural OAD at risk of mental health symptoms.

